# Transcranial burst electrical stimulation contributes to neuromodulatory effects in the rat motor cortex

**DOI:** 10.3389/fnins.2023.1303014

**Published:** 2023-12-11

**Authors:** Thi Xuan Dieu Nguyen, Chi-Wei Kuo, Chih-Wei Peng, Hao-Li Liu, Ming-Yuan Chang, Tsung-Hsun Hsieh

**Affiliations:** ^1^School of Physical Therapy and Graduate Institute of Rehabilitation Science, Chang Gung University, Taoyuan, Taiwan; ^2^School of Biomedical Engineering, College of Biomedical Engineering, Taipei Medical University, Taipei, Taiwan; ^3^Department of Electrical Engineering, National Taiwan University, Taipei, Taiwan; ^4^Division of Neurosurgery, Department of Surgery, Min-Sheng General Hospital, Taoyuan, Taiwan; ^5^Healthy Aging Research Center, Chang Gung University, Taoyuan, Taiwan; ^6^Neuroscience Research Center, Chang Gung Memorial Hospital, Taoyuan, Taiwan

**Keywords:** transcranial burst electrical stimulation, neuromodulation, motor evoked potential, neuroplasticity, rats

## Abstract

**Background and objective:**

Transcranial Burst Electrical Stimulation (tBES) is an innovative non-invasive brain stimulation technique that combines direct current (DC) and theta burst stimulation (TBS) for brain neuromodulation. It has been suggested that the tBES protocol may efficiently induce neuroplasticity. However, few studies have systematically tested neuromodulatory effects and underlying neurophysiological mechanisms by manipulating the polarity of DC and TBS patterns. This study aimed to develop the platform and assess neuromodulatory effects and neuronal activity changes following tBES.

**Methods:**

Five groups of rats were exposed to anodal DC combined with intermittent TBS (tBES+), cathodal DC combined with continuous TBS (tBES−), anodal and cathodal transcranial direct current stimulation (tDCS+ and tDCS−), and sham groups. The neuromodulatory effects of each stimulation on motor cortical excitability were analyzed by motor-evoked potentials (MEPs) changes. We also investigated the effects of tBES on both excitatory and inhibitory neural biomarkers. We specifically examined c-Fos and glutamic acid decarboxylase (GAD-65) using immunohistochemistry staining techniques. Additionally, we evaluated the safety of tBES by analyzing glial fibrillary acidic protein (GFAP) expression.

**Results:**

Our findings demonstrated significant impacts of tBES on motor cortical excitability up to 30 min post-stimulation. Specifically, MEPs significantly increased after tBES (+) compared to pre-stimulation (*p* = 0.026) and sham condition (*p* = 0.025). Conversely, tBES (−) led to a notable decrease in MEPs relative to baseline (*p* = 0.04) and sham condition (*p* = 0.048). Although tBES showed a more favorable neuromodulatory effect than tDCS, statistical analysis revealed no significant differences between these two groups (*p* > 0.05). Additionally, tBES (+) exhibited a significant activation of excitatory neurons, indicated by increased c-Fos expression (*p* < 0.05), and a reduction in GAD-65 density (p < 0.05). tBES (−) promoted GAD-65 expression (*p* < 0.05) while inhibiting c-Fos activation (*p* < 0.05), suggesting the involvement of cortical inhibition with tBES (−). The expression of GFAP showed no significant difference between tBES and sham conditions (*p* > 0.05), indicating that tBES did not induce neural injury in the stimulated regions.

**Conclusion:**

Our study indicates that tBES effectively modulates motor cortical excitability. This research significantly contributes to a better understanding of the neuromodulatory effects of tBES, and could provide valuable evidence for its potential clinical applications in treating neurological disorders.

## 1 Introduction

In recent decades, there has been a growing interest in non-invasive brain stimulation (NIBS) techniques that have demonstrated the ability to modify brain activity and modulate cortical plasticity. These techniques can be employed individually or in conjunction with other rehabilitative therapies to enhance training effectiveness (Schulz et al., 2013; Cirillo et al., 2017; Polanía et al., 2018; Sanches et al., 2020; Grippe et al., 2022). Currently, available NIBS methods include repetitive transcranial magnetic stimulation (rTMS), transcranial direct current stimulation (tDCS), transcranial alternating current stimulation (tACS), random noise stimulation (tRNS), and transcranial ultrasound stimulation (TUS; Terranova et al., 2018; Bhattacharya et al., 2022). Nevertheless, among these techniques, rTMS and tDCS remain the most frequently used in clinical settings for the treatment of neurological diseases and disorders. These NIBS have received substantial research support and have yielded positive results, such as reducing sleep disturbances in neurological and neuropsychiatric conditions, enhancing cognitive functions in Alzheimer’s disease, aiding in the recovery of upper and lower limb functions in stroke patients, and improving both motor and non-motor symptoms in patients with Parkinson’s disease, among others (Fregni and Pascual-Leone, 2007; Cirillo et al., 2017; Santos Ferreira et al., 2019; Begemann et al., 2020; Sanches et al., 2020; Chu et al., 2021; Herrero Babiloni et al., 2021; Madrid and Benninger, 2021; Parikh et al., 2021; Semmler et al., 2021; Veldema and Gharabaghi, 2022).

Regarding tDCS, a weak direct electric current (usually less than 2 mA) is applied to the brain using two or more electrodes positioned on the scalp. tDCS generates currents between the anode and cathode electrodes, resulting in polarity-specific changes in the resting membrane potential of neuronal cells, producing the desired effect (Morya et al., 2019; Santos Ferreira et al., 2019; Lefaucheur et al., 2020; Chan et al., 2021). tDCS offers several practical advantages over rTMS, as it is safer (not associated with seizures; Bikson et al., 2016), more cost-effective, easier to administer, may even be used as a home-based medical device (Charvet et al., 2015). In contrast, repetitive transcranial magnetic stimulation (rTMS) stimulates the target brain area using a magnetic coil to induce a magnetic field. rTMS can be applied at different frequencies, including low (≤1 Hz) and high (>1 Hz) frequencies (Lefaucheur et al., 2020). Among various rTMS protocols, theta burst stimulation (TBS) induced by either TMS or electrical stimulation is a recent pattern designed to mimic hippocampal theta rhythms. These rhythms have been demonstrated to induce long-term potentiation/depression-like effects (Nguyen and Kandel, 1997; Paulus, 2005; Kouvaros and Papatheodoropoulos, 2016; Cacace et al., 2017). TBS generates more powerful and longer-lasting effects compared to previous protocols. It consists of bursts of three pulses delivered at 50 Hz, repeated every 200 ms, and lasting for 2 s. TBS is administered in two forms: intermittent TBS (iTBS), involving a 2-s train of bursts repeated every 10 s, and continuous TBS (cTBS), where a continuous train of bursts is delivered. iTBS generates an excitatory effect, while cTBS induces an inhibitory effect (Huang et al., 2005; Rounis and Huang, 2020).

By integrating the advantages of tDCS with the effective patterns of TBS, a novel NIBS protocol known as transcranial burst electrical stimulation (tBES) has attracted considerable attention. The innovative tBES protocol, which combines direct current (DC) with TBS-like waveforms, was proposed and has shown preliminary findings indicating that the combination of anodal DC with an intermittent theta burst stimulation (iTBS) waveform may enhance the effectiveness of brain neuroplasticity (Li et al., 2019). More recent studies suggest that applying tBES via conventional or high-definition (HD) electrodes targeting the affected hemisphere has yielded positive neurorehabilitation outcomes in upper limb function among stroke patients (Chen et al., 2021; Huang Y. J. et al., 2022). The potential of tBES waveforms could be a promising neuromodulatory approach for further neurological treatment. However, the complete tBES protocols, including facilitative and inhibitive waveforms for inducing neuromodulation effects and the related underlying neurophysiological mechanisms, remain to be fully elucidated.

This study, conducted at the bench stage of translational research, aimed to investigate the neuromodulatory effects of tBES on cortical motor activation by analyzing the variability of motor-evoked potentials (MEPs). To investigate the underlying neurophysiological mechanisms, particularly the NIBS-induced changes in neuronal activity, previous studies utilized immediate-early genes such as c-Fos or GAD-65 as markers of excitatory and inhibitory neuronal activity in the brains of rodents that had undergone rTMS (Trippe et al., 2009; Moretti et al., 2022). Therefore, we also examined the impact of tBES on excitatory and inhibitory neural biomarkers, specifically assessing c-Fos (an immediate-early gene) and glutamic acid decarboxylase (GAD-65) through immunohistochemistry staining. Finally, we assessed the safety of tBES by analyzing glial fibrillary acidic protein (GFAP) expression in astrocytes. We hypothesized that tBES can significantly modulate cortical excitability within the stimulated region compared to sham stimulation and enhance the corresponding neural biomarkers, and show no differences in GFAP expression compared to the sham group.

## 2 Materials and methods

### 2.1 Animal preparation and experimental design

**Figure 1:**
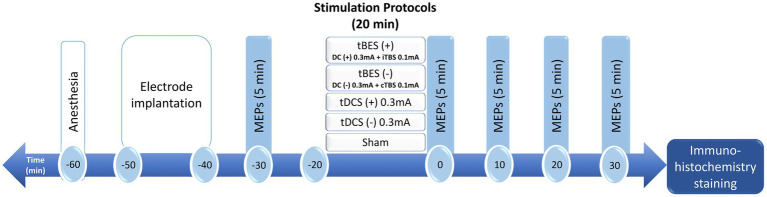
Schematic diagram of the experimental design for testing changes in cortical excitability after transcranial burst electrical stimulation (tBES), transcranial direct current stimulation (tDCS) or sham stimulation in anesthetized rats. In this representative experiment, anesthetized rats received intervention protocols for a duration of 20 min. Motor-evoked potentials (MEPs) were measured at baseline and at 0, 10, 20, and 30 min following the intervention. Subsequently, the rat brains were removed and subjected to immunohistochemistry for further investigation. DC, direct current; iTBS, intermittent theta burst stimulation; cTBS, continuous theta burst stimulation.

All animal experiments were conducted in accordance with the guidelines and were approved by the Institutional Animal Care and Use Committee of Chang Gung University (IACUC No. CGU108-202). Forty male adult Sprague–Dawley rats (280–320 g; BioLASCO, Taipei, Taiwan) were utilized for the experiments. These rats were individually housed in standard cages with unrestricted access to food and water, and they were maintained in a temperature-controlled environment with a 12-h light/dark cycle prior to the commencement of the experiment. Every effort was made to minimize the use of rats in this study. The rats were randomly assigned to one of five groups (tBES+, tBES−, tDCS+, tDCS−, and sham), each consisting of eight rats. These rats received electrical stimulation protocols and underwent electrophysiological recordings under anesthesia. The experimental design is illustrated in Figure 1.

### 2.2 Experimental setup

**Figure 2:**
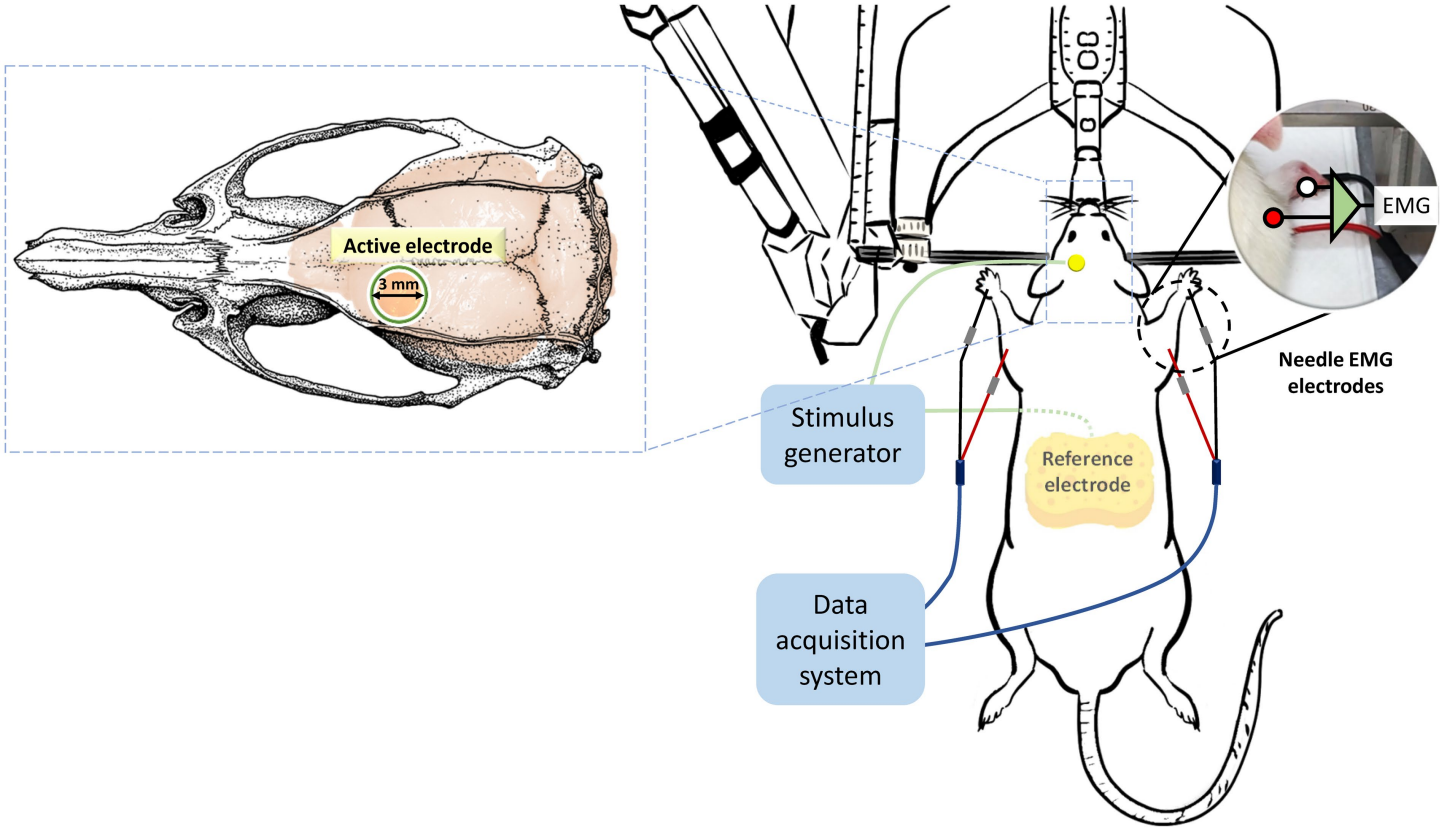
Placement and assembly of stimulation and recording electrodes. Throughout the entire experimental period, the rats were securely mounted on a stereotactic apparatus. Stimulation protocols were administered using an active electrode positioned 2.5 mm laterally and 1.5 mm anterior to the bregma, while a reference electrode was placed in the abdominal region. The same electrode configurations were employed to elicit motor-evoked potentials (MEPs). MEP data, recorded from the brachioradialis muscles, were subsequently analyzed to assess alterations in cortical excitability resulting from the intervention protocols.

On the day of the experiment, the rat was deeply anesthetized for approximately 2.5 h with an intraperitoneal injection (i.p.) of tiletamine-zolazepam (50 mg/kg; Zoletil, Vibac, France) and xylazine (10 mg/kg; Rompun, Bayer, Germany). Once the rat lost its toe-pinch reflex, it was securely positioned on a stereotaxic frame. Subsequently, the scalp and tissue were carefully removed and cleaned, exposing the bregma line to identify the electrode’s location. To administer the intervention protocols and deliver the stimuli to the motor cortex, a circular plastic socket with an inner diameter of 3 mm was affixed to the skull, precisely above the left primary motor cortex, corresponding to the right forelimb. This attachment was achieved using cyanoacrylate glue and dental cement. The center of this socket was positioned at coordinates 2.5 mm laterally and 1.5 mm anteriorly from the bregma point, focusing on the motor cortex of the unilateral forelimb in rats, as determined by stereotactic measurements (Fonoff et al., 2009). Immediately before stimulation, the socket was filled with conductive gel, and a 0.2 mm diameter silver wire, serving as the active electrode, was connected to an electrical stimulator (STG4002, Multichannel Systems, Reutlingen, Germany). The reference electrode consisted of a saline-soaked sponge placed within the abdomen (7 cm × 5 cm, EASYpadTM, Soterix Medical Inc., NY, United States; Figure 2).

### 2.3 Stimulation protocols

**Figure 3:**
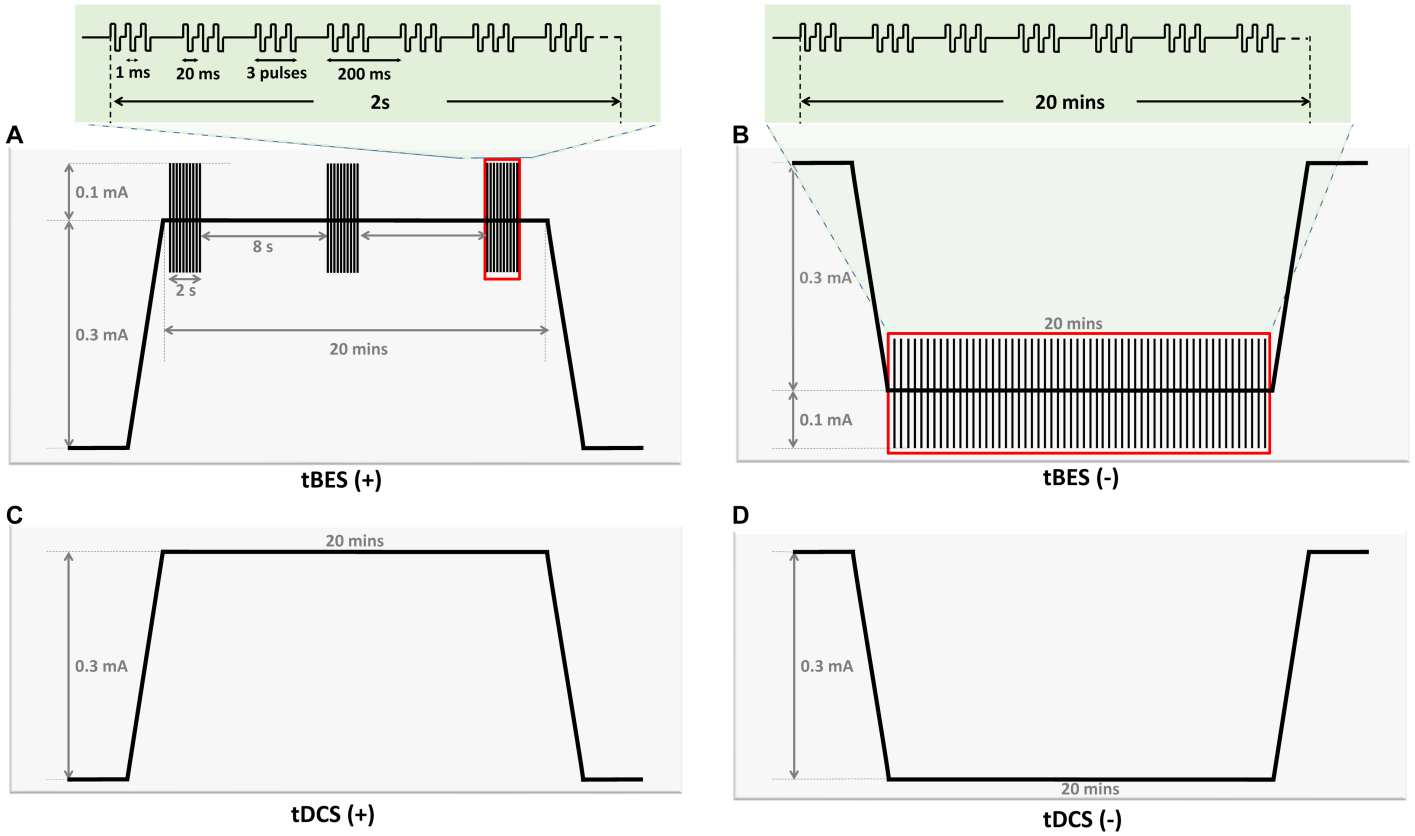
Graph illustrating parameters of four stimulation protocols in the current study. The graph provides an overview of the parameters for the four stimulation protocols utilized in this study. tBES (+): This protocol combines tDCS (+) with intermittent theta burst (iTBS) mode. iTBS comprises 10 bursts of three pulses delivered at a frequency of 50 Hz, with each burst lasting 200 ms and repeated for 2 s, followed by an 8-s rest **(A)**. tBES (−): In this protocol, tDCS (−) is paired with continuous theta burst (cTBS) mode, consisting of bursts of three pulses delivered at a frequency of 50 Hz, with each burst lasting 200 ms **(B)**. tDCS (+): Anodal direct current stimulation is applied at an intensity of 0.3 mA **(C)**. tDCS (−): Cathodal direct current stimulation is applied at an intensity of 0.3 mA **(D)**. All stimulation protocols were administered for 20 min.

The stimulation protocols were programmed using Multichannel Stimulus II software, compatible with a two-channel electrical stimulator (STG4002, Multichannel Systems, Reutlingen, Germany). Figure 3 presents the details of all the stimulation protocols. Traditional tDCS and Theta Burst Stimulation (TBS) protocols are widely used neuromodulation approaches for various neurological disorders (Bologna et al., 2015; Bornheim et al., 2022; Huang W. et al., 2022; Zhang et al., 2022). For example, anodal tDCS increases neuronal excitability, producing long-term potentiation (LTP)-like effects, while cathodal tDCS induces long-term depression (LTD)-like effects (Rroji et al., 2015; Kronberg et al., 2017). Additionally, continuous TBS (cTBS) induces LTD-like cortical plasticity, whereas intermittent TBS (iTBS) produces LTP-like effects (Kirkovski et al., 2023). In this study, the tBES pattern combines both facilitation and inhibition waveforms from direct current and TBS. The tBES (+) and tBES (−) waveforms were selected to investigate whether the tBES protocols may yield synergistic and higher neuromodulatory effects. Therefore, five stimulus protocols, including tBES (+), tBES (−), tDCS (+), tDCS (−), or the sham protocol, were designed. For the tBES (+) and tBES (−) groups, the intensity was set at 0.3 mA of direct current combined with 0.1 mA of theta burst. For tDCS, a constant anodal or cathodal direct current of 0.3 mA, with a charge density of 5.1 C/cm^2^ and a current density of 4.2 mA/cm^2^, was delivered to healthy rats for 20 min. The current density of these tDCS protocols was reported to induce no damage to brain tissues (Bikson et al., 2016; Chhatbar et al., 2017). The sham group received a fixed current amplitude of 0 mA. Each stimulation session was performed for 20 min.

### 2.4 Assessments of cortical excitability

The electrophysiological recording was conducted after 30 min anesthesia to ensure that the anesthesia had reached a stable condition (Hsieh et al., 2015; Liu et al., 2019). Furthermore, to confirm no confounding effects of anesthesia, MEPs were recorded at multiple time points in the sham-stimulation group. For measuring MEPs, the electromyography (EMG) data was recorded using 27G stainless steel needle electrodes (Axon Systems Inc., Hauppauge, NY, United States) inserted into the left and right brachioradialis muscles, with the reference electrode positioned distally in the paw. The ground electrode was inserted at the base of the rat’s tail. The EMG data were subsequently amplified (gain ×1,000) and filtered (notch 60 Hz and bandpass 10–1,000 Hz) before digitization at a sampling rate of 10 kHz using a data acquisition system (MP36, BIOPAC system, California, United States). The recorded data were saved for offline analyses. The same epicranial stimulation electrode montages were used to deliver single stimuli for measuring cortical excitability through MEPs. Stimulation was applied to the unilateral motor cortex, and the magnitude of the evoked muscle contractions in both contralateral and ipsilateral limbs was assessed. Regarding MEP recording, single biphasic pulses with a pulse duration of 1 ms and 10-s intervals were initially delivered to determine the Resting Motor Threshold (RMT). RMT was defined as the minimum intensity of electrical stimulation required to elicit peak-to-peak MEPs greater than 20 μV in five out of 10 trials. Subsequently, MEPs were collected continuously every 10 s at an intensity of 120% of RMT for 5 min. MEP recordings were conducted at specific time points, including before the intervention stimulation (baseline), immediately after the intervention, and at 10-min intervals up to 30 min after the intervention stimulation ended (post 0, 10, 20, and 30 min, respectively; Figure 1).

### 2.5 Immunohistochemistry staining

After conducting electrophysiological assessments, we transcardially perfused rats with saline, followed by ice-cold 4% paraformaldehyde (PFA) in 0.1 M phosphate-buffered saline (PBS). The rat brains were then post-fixed in a 4% PFA solution for 2 days and cryoprotected in a 30% sucrose solution at 4°C overnight until they sank. Subsequently, the brains were coronally sectioned into serial sections with a thickness of 30 μm using a cryostat after being frozen at −81°C (Leica CM3050S Cryostat, FL, United States). Selected sections were incubated in a blocking solution (PBS with 10% goat serum) at room temperature for 1 h, following the inhibition of endogenous antigen activity with 0.3% H2O2 in PBS for 10 min. Afterward, these sections were exposed to primary rabbit antibodies against GFAP (1:1,000, AB7260, Millipore, United States), c-Fos (1:1,000, AB11959, Millipore, United States), and GAD-65 (1:100, AB239372, Millipore, United States) for 1 h at room temperature, followed by secondary anti-rabbit antibodies (1:200, MP-7401, Vector Labs, United States) for 1 h at room temperature. The sections were washed three times before adding a solution of 3.3-diaminobenzidine (DAB, SK-4105, Vector Labs, United States) for 10 min, and then all markers were visualized under specific colors.

Finally, using a digital pathology slide scanner (Aperio CS2, Leica Biosystems Inc., Buffalo Grove, IL, United States), images of regions of interest (ROI) on slide-mounted sections were captured at 40X optical zoom (0.25 M/pixel). Quantitative analysis of GFAP, c-fos, and GAD-65 was carried out manually and automatically to determine the level of expressed cells. Cell counting was performed on images obtained from the Aperio ImageScope viewer program at high magnification. A defined threshold for specific cells in the ROI, consistent across all images, was applied, and the resulting images were converted to black and white (BW). Next, cell density in specific regions was measured using computer-based image analysis software (Image-Pro, Media Cybernetics, Bethesda, MD, USA). Two researchers then thoroughly examined the results to ensure the correct identification of immunoreactivity patterns. Finally, the density of expressing cells was calculated within each ROI and expressed as the mean number of cells per mm^2^ (cells/mm^2^).

### 2.6 Data and statistical analysis

The peak-to-peak amplitudes of the MEPs were processed using MATLAB R2021a version (The MathWorks Inc., United States), and the average amplitudes of 30 consecutive MEPs were manually determined. Before performing statistical analysis, we normalized the averaged MEP amplitudes at each post-intervention time point to the averaged baseline MEP amplitude. Next, we proceeded to compare MEP amplitudes between groups utilizing a two-way repeated measures analysis of variance (ANOVA) conducted in SPSS version 22.0 for Windows (SPSS Inc., United States). The within-subject factor was TIME, with five levels: pre-intervention, 0, 10, 20, and 30 min after interventions, across five intervention protocols. If significant main effects and interactions were found, we performed independent *t*-tests to compare the groups at each time point. We also assessed the time course of changes in each protocol on the absolute amplitude values of MEPs using a separate one-way ANOVA, followed by post-hoc Fisher’s LSD tests to determine differences between time points. Additionally, comparing immuno-histochemical data between and within groups required independent and paired *t*-tests. All data were presented using the mean and standard error of the mean (SEM), and we established a value of *p* cutoff of 0.05 for statistical significance.

## 3 Results

### 3.1 Neuromodulatory effects

When comparing the MEP responses in the contralateral limb (right limb) among the intervention protocols, the two-way repeated ANOVA did not reveal a significant main effect of time [*F*(2.776; 97.152) = 1.531, *p* = 0.214]. However, there were substantial differences between groups [*F*(4;35) = 8.570, *p* < 0.001] and a significant interaction [TIME × GROUP: *F*(11.103; 97.152) = 3.986, *p* < 0.001], indicating significant differences in MEP amplitudes between groups and over time within each group. Subsequently, we conducted a one-way ANOVA to evaluate differences within each group at each time point and an independent t-test to compare across groups at each time point.

**Figure 4:**
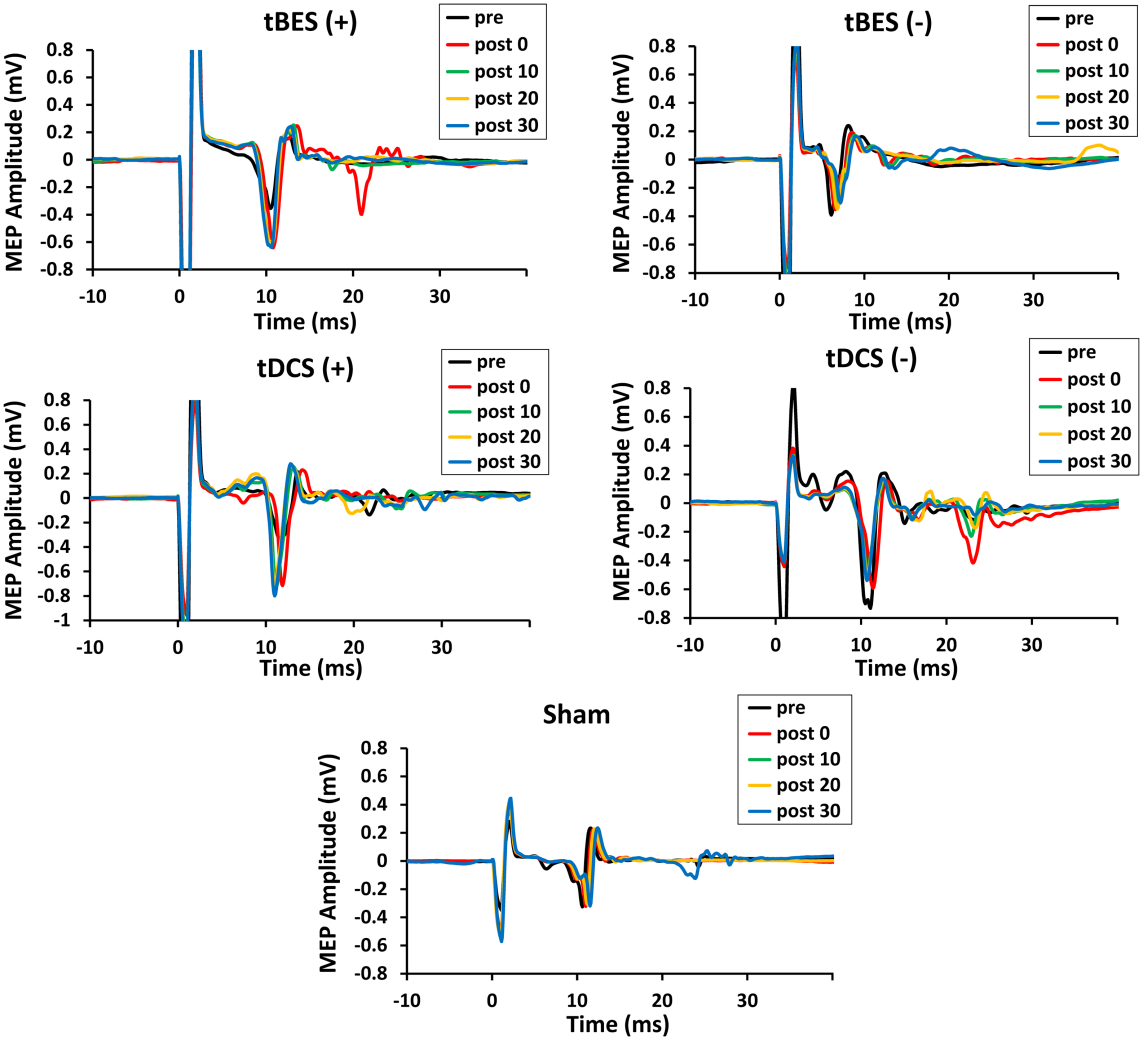
The time course changes in raw motor-evoked potential (MEP) signals for tBES (+), tDCS (+), tBES (−), tDCS (−), and the sham condition. MEP amplitudes increased after tBES (+) and tDCS (+), while they decreased following tBES (−) and tDCS (−) over the 30-min post-intervention period compared to baseline. No significant changes in MEP signals were observed under the sham condition.

Figure 4 illustrates the time course changes in raw MEPs produced under each intervention protocol. Both tBES (+) and tDCS (+) resulted in MEP amplitude facilitation at all time points, with tBES (+) generally having a more robust effect than tDCS (+), although there was no statistically significant difference in averaged MEP amplitudes between these two treatments (*p* > 0.05).

**Figure 5:**
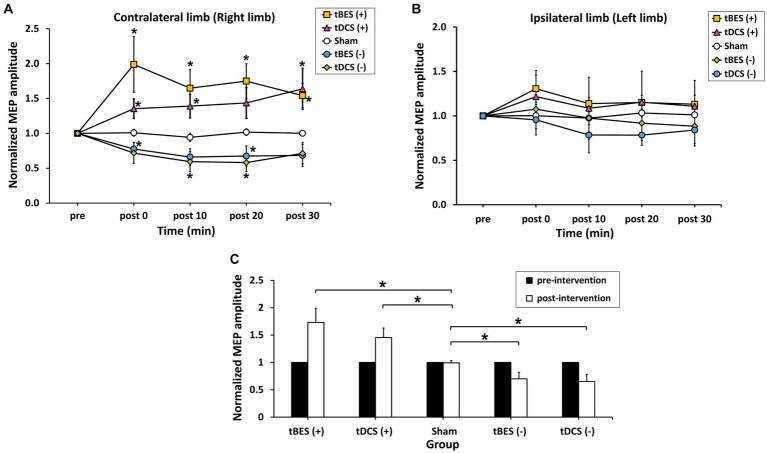
The average normalized motor-evoked potential (MEP) amplitudes for the contralateral limb **(A)** and ipsilateral limb **(B)** across the five intervention protocols (tBES+, tDCS+, tBES−, tDCS−, and sham). The averaged responses of cortical excitability were calculated for each intervention group within 30 min following different stimulations on the contralateral limb **(C)**. Asterisks (*) indicate statistically significant differences when comparing tBES (+), tBES (−), tDCS (+), and tDCS (−) with the sham group at the same time point. The data are presented as means, with error bars representing the standard error of the mean (SEM); ^*^p ≤ 0.05.

Notably, MEP amplitudes significantly increased after tBES (+) compared to baseline, although this increase slightly decreased at 10-, 20-, and 30-min post-stimulation; significant differences were identified for all time periods compared to the sham group at the same time points (*p* < 0.05). Similarly, tDCS (+) had an apparent influence on MEPs, with a statistical difference observed at post-0 (*p* = 0.048) and 10-min (*p* = 0.043) time points compared to the sham stimulation group (Figure 5A). In terms of MEP suppression, both tBES (−) and tDCS (−) resulted in reduced MEP amplitudes compared to the sham stimulation group and pre-intervention baseline. However, at specific time points (post 10- and post 30-min for tBES−; post 0- and post 30-min for tDCS−), statistical analyses did not reveal significant variations in mean MEP amplitudes. Additionally, there were no discernible differences in MEPs between the two intervention protocols (*p* > 0.05).

Additionally, MEPs showed varied in the ipsilateral limb (left limb) after stimulation (Figure 5B). Statistical analyses on the MEP data did not reveal any significant main effects [TIME: *F*(2.528; 88.470) = 0.679, *p* = 0.543; GROUP: *F*(4; 35) = 0.929, *p* = 0.458], nor did they show a significant interaction between time and group [TIME × GROUP: *F*(10.111; 88. 470) = 0.722, *p* = 0.703]. It indicates that MEPs obtained from the ipsilateral limb were not markedly affected by contralateral stimulation.

To compare the neuromodulatory effects among the different stimulation groups, we averaged the longitudinal MEP responses over 30 min. Figure 5C displays averaged level of MEPs at pre- and across 30 min post-tBES (+), tDCS (+), tBES (−), tDCS (−), and sham stimulation. The average MEP responses to tBES (+), tBES (−), tDCS (+), and tDCS (−) were significantly larger than those to the sham stimulation, confirming that four intervention regimens had substantial impacts on the overall changes in mean MEPs.

### 3.2 Histological assays

To confirm whether tBES indeed activates neurons and GABAergic synaptic terminals, as indicated by the increase in immediate early genes c-Fos and GAD-65, respectively, we conducted experiments on 12 rats divided into three groups: tBES (+), tBES (−), and sham, with four rats in each group. These rats received the stimulation protocols and were subsequently transcardially perfused after 20 min of stimulation, and their brains were subjected to immunohistochemistry (IHC) analysis.

**Figure 6:**
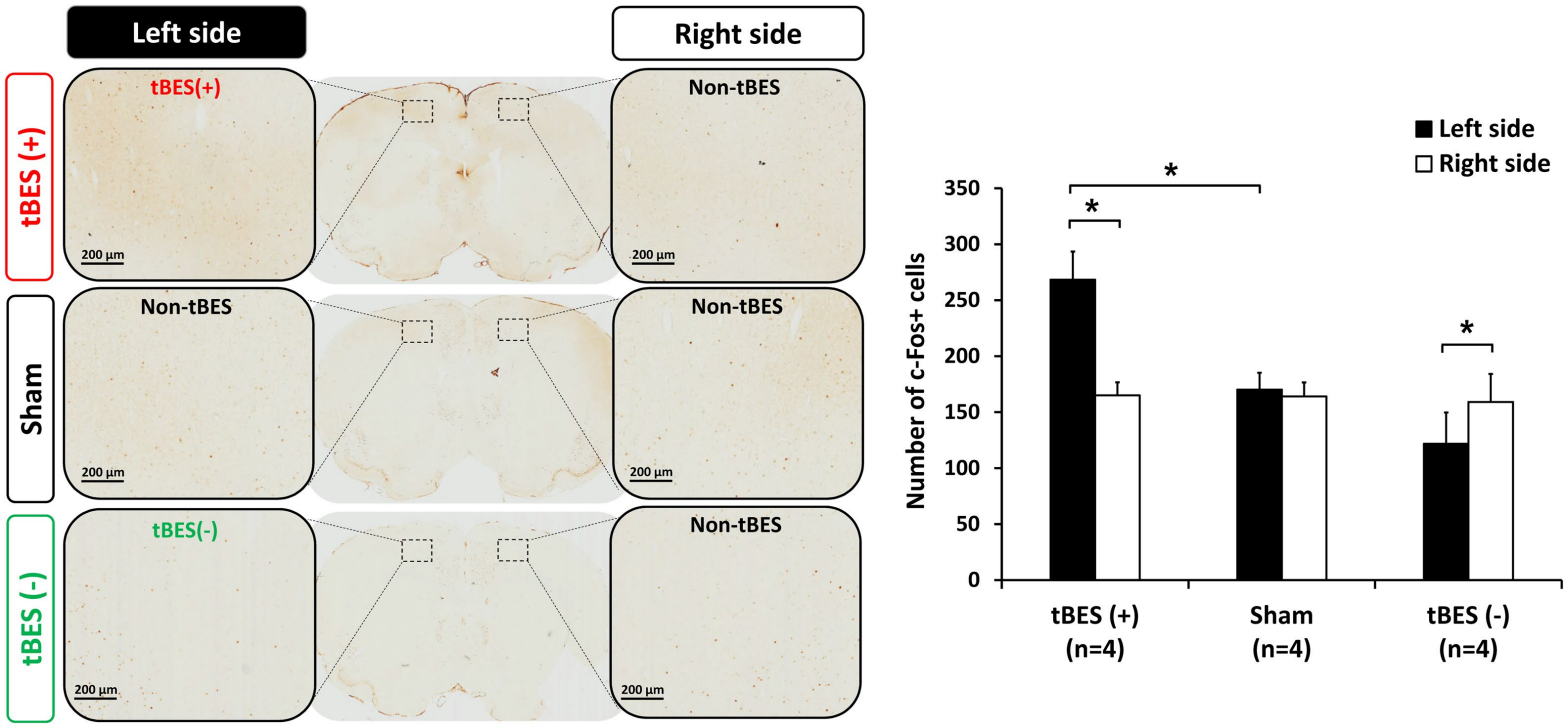
Representative immunohistochemically stained slices with regions of interest (ROI) **(A)**. Data for c-Fos are presented as the number of labeled cells within the ROI. The changes in cortical expression of c-Fos following tBES (+), tBES (−), and sham stimulation **(B)**. Asterisks (*) indicate statistically significant differences, either between the right and left sides of the brain within a group or when compared to the sham group on the same side of the brain. The data are presented as means, with error bars representing the standard error of the mean (SEM); ^*^p ≤ 0.05.

Our findings revealed that tBES (+) significantly increased the number of c-Fos positive cells in the stimulated region, specifically the left side of the motor cortex, at 20 min post-stimulation (Figure 6A). This increase was statistically significant when compared to both the contralateral motor cortex in the tBES (+) group (*p* = 0.012) and the sham group (*p* = 0.015). In contrast, following tBES (−) stimulation, we observed a decrease in c-Fos expression compared to sham stimulation and tBES (+) on the same side and compared to the right side of the motor cortex. However, a statistically significant difference was only found within the tBES (−) group (*p* = 0.05), not between the tBES (−) and sham groups. Significantly, sham stimulation did not affect the expression of c-Fos on either side of the motor cortex (Figure 6).

**Figure 7:**
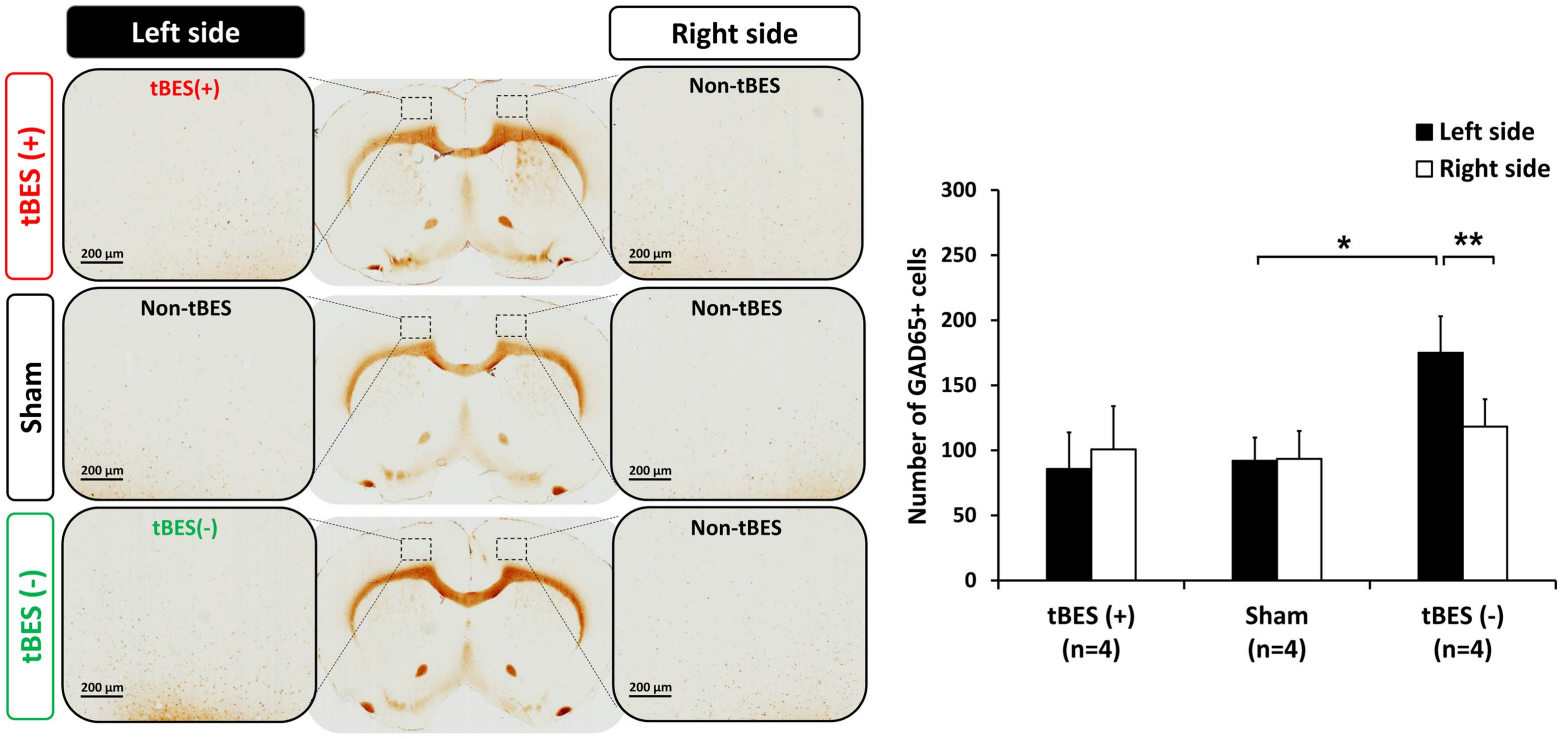
Representative images of GAD-65 immunostaining **(A)** and the average changes in GAD-65 expression following tBES (+), tBES (−), and sham stimulation **(B)**. Asterisks (*) indicate statistically significant differences between the right and left sides of the brain within a group or when compared to the sham group on the same side of the brain. The data are presented as means, with error bars representing the standard error of the mean (SEM); ^**^p ≤ 0.05, ^*^p ≤ 0.01.

Regarding the inhibitory neurobiological marker, after tBES (−) stimulation, we observed an elevation in GAD-65 expression in the stimulated region but not in the contralateral region (*p* = 0.004). Furthermore, the quantity of GAD-65+ cells in the tBES (−) group was greater than that in the sham group when compared to the sham group in the same hemisphere (*p* = 0.047; Figure 7). Our results confirm that tBES (+) and tBES (−), but not sham stimulation, clearly activate excitatory and inhibitory neurons, respectively.

**Figure 8:**
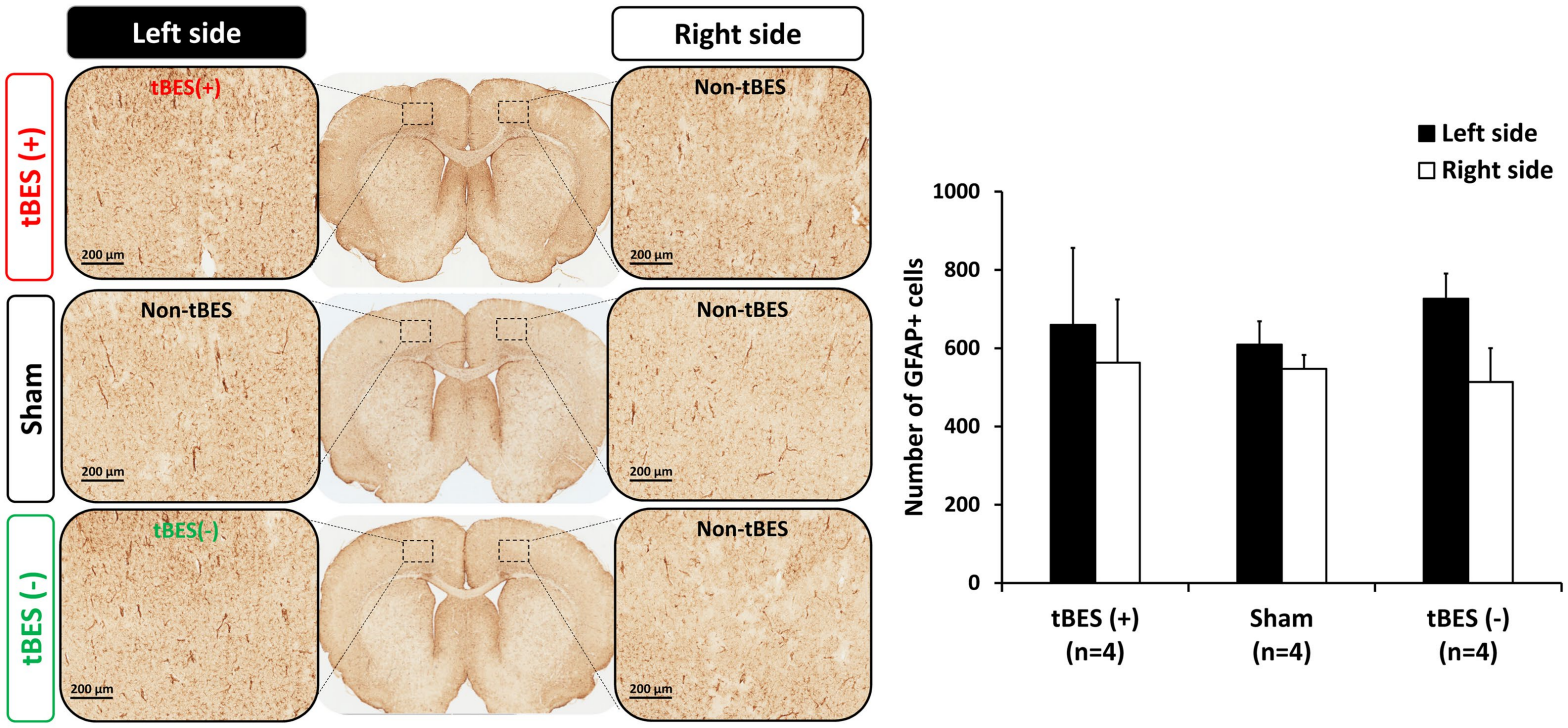
Representative images of glial fibrillary acidic protein (GFAP) immunostaining **(A)** and the average changes in GFAP expression following tBES (+), tBES (−), and sham stimulation **(B)**. Asterisks (*) indicate statistically significant differences either between the right and left sides of the brain within a group or when compared to the sham group on the same side of the brain. Data are presented as means, with error bars representing the standard error of the mean (SEM).

We then further assessed the safety of tBES by examining the expression of GFAP-immunoreactive astrocytes, which are known to become activated in response to brain damage (Luoto et al., 2017). Immunohistochemistry (IHC) staining was performed to detect GFAP-positive cells 24 h after stimulation. Our IHC analyses revealed a slight increase in the density of GFAP-positive cells in rats subjected to either tBES (+) or tBES (−) compared to the non-stimulation region in both the tBES and sham groups. However, these changes were not statistically significant (all *p* > 0.05; Figure 8).

## 4 Discussion

The field of neuromodulation is continuously evolving, with a focus on enhancing treatment efficacy. Innovative techniques based on NIBS methods that are already in use are being developed (Terranova et al., 2018; Antal et al., 2022). The recent introduction of tBES may help bridge existing gaps and provide therapists with an alternative for treating neurological disorders. Since tBES combines direct current and theta burst waveforms, it is expected to have a superior impact on neural plasticity and offer therapeutic benefits for neurological diseases such as stroke, Alzheimer’s, and Parkinson’s disease.

In the current study, we aimed to identify and compare the effects of tBES and pure tDCS on cortical excitability using a rodent model. We performed unilateral cortical motor stimulation using a plastic socket filled with electrolyte and an electrode, similar to a human experiment setup. To validate the neuromodulatory effects of tBES, we assessed MEPs, which reflect long-term potentiation (LTP)-like and long-term depression (LTD)-like plasticity in the motor cortex (Delvendahl et al., 2012). Based on the analysis of contralateral MEP data, our findings support the hypothesis that tBES (+) significantly increases motor cortex excitability. This increase was greater than that produced by sham stimulation and pure tDCS (+). On the other hand, the tBES (−) group exhibited a more significant reduction in cortical excitability than the sham (−) group but did not differ significantly from the pure tDCS (−) group. Additionally, our results indicate that tBES-induced MEPs in the contralateral limb were larger than those in the ipsilateral limb, suggesting that tBES may have a targeted effect on the desired cortical region. While our study did not include behavioral tests following stimulation protocols, it is worth noting that MEPs are often interpreted in the context of motor function execution, suggesting a potential correlation between tBES-induced MEPs and changes in motor function (Bestmann and Krakauer, 2015).

In addition to investigating the mechanisms underlying tBES’s promotion of LTP- or LTD-like plasticity through NMDA or GABA receptor activation, we examined the density of neural cells, specifically c-Fos, and GAD-65, within motor cortical areas. The NMDA receptor, an essential glutamate receptor, plays a crucial role in LTP and synaptic plasticity by involving key molecules like calcium ions, PSD-95, CaMK II, PKA, MAPK, CREB, and immediate early genes (IEGs) in the NMDA signaling pathway (Brigman et al., 2010; Wang and Peng, 2016). Among these, c-Fos is an IEG whose expression is positively correlated with the induction of LTP (Abraham et al., 1991; Lüscher and Malenka, 2012; Wang and Peng, 2016). c-Fos is also a valuable marker for identifying excited neurons following stimulation, providing insight into the neuronal mechanisms of treatment effects (Doi et al., 2001; Zhang et al., 2002; Hoppenrath and Funke, 2013; Li et al., 2015). Previous research has shown that anodal tDCS increases c-Fos expression in both the primary motor cortex and substantia nigra (SN) in Parkinson’s disease monkeys (Li et al., 2015). On the other hand, GABA is the primary inhibitory neurotransmitter, responsible for reducing neuronal excitability (Wong et al., 2003). The activity and expression of GAD are closely linked to GABA levels and subsequent inhibitory neurotransmission at synapses (Lee et al., 2019). GAD-65 expression in cortical inhibitory interneurons is often assessed after NIBS (Trippe et al., 2009; Hoppenrath and Funke, 2013). GABA is synthesized by GAD-65 in an activity-dependent manner, contributing to the balance between excitatory and inhibitory mechanisms (Müller et al., 2015; Lee et al., 2019; Kajita and Mushiake, 2021). Earlier studies have indicated that GAD-65 expression decreases following anodal tDCS (Heimrath et al., 2020; Zhao et al., 2020; Yamada and Sumiyoshi, 2021). Consistent with previous NIBS research on excitatory and inhibitory mechanisms, our findings demonstrate that tBES (+) enhances the synthesis of NMDA receptors, as indicated by upregulated c-Fos and downregulated GAD-65 expression. In contrast, tBES (−) increases the number of GAD-65 cells and decreases the number of c-Fos cells, suggesting that GABA receptors may be abundantly activated under tBES (−). These results align with those of MEPs at the same time points.

GFAP, responsible for providing structural stability to astrocyte processes, plays a crucial role in modulating astrocyte mobility and morphology. When brain damage occurs, astrocytes become reactive, rapidly synthesize GFAP, and respond (Eng et al., 2000). Numerous *in vivo* and *in vitro* studies have shown that regulating GFAP in astrocytes is not only beneficial for understanding the physiology of healthy brains but also serves as a biomarker for neurological diseases like Alzheimer’s and Parkinson’s disease (Gomes et al., 1999; Pekny and Pekna, 2004; Middeldorp and Hol, 2011). We evaluated GFAP expression to assess whether the motor cortex was damaged following tBES. Our results show that tBES upregulates GFAP expression 24 h after tBES, but not significantly. This increase may be explained by our choice of investigating GFAP expression 24 h after stimulation, as GFAP has been shown to peak at this time and then rapidly decline afterward (Fujiki and Steward, 1997).

We acknowledge several limitations in our study. First, we assessed MEP alterations in short-term after-effects, which may fluctuate over a longer-term observation with additional time points beyond the 30-min post-stimulation protocols. Thus, future research could explore the longer-term after-effects of tBES, potentially extending the measurement period to an hour or beyond. Second, we examined the safety of tBES in rats that underwent intervention protocols and MEP recordings. Although we investigated GFAP expression on both sides of the brain and used a sham group, it is possible that single pulses used to generate MEPs might impact the significant increase in the number of GFAP expressions within the stimulated area. Furthermore, we did not measure other neural activity indicators or additional parameters to definitively determine the neurophysiological mechanisms of tBES efficacy. To address this limitation, further studies should consider investigating changes in synaptic plasticity, neurotransmitter levels, or other relevant outcome measures to provide a more comprehensive understanding of the neuromodulatory effects of tBES. In conclusion, our study highlights the potential of tBES as a safe neuromodulation technique capable of inducing neural plasticity and activating neural cells. The relevance of our findings to human and disease models should be validated in future studies.

## Data availability statement

The original contributions presented in the study are included in the article/Supplementary material, further inquiries can be directed to the corresponding author.

## Ethics statement

The animal study was approved by Institutional Animal Care and Use Committee of Chang Gung University (IACUC No. CGU108-202). The study was conducted in accordance with the local legislation and institutional requirements.

## Author contributions

TXDN: Conceptualization, Data curation, Formal analysis, Investigation, Methodology, Writing – original draft, Writing – review & editing. C-WK: Data curation, Formal analysis, Investigation, Methodology, Writing – original draft, Writing – review & editing. C-WP: Resources, Writing – original draft, Writing – review & editing. H-LL: Resources, Writing – original draft, Writing – review & editing. M-YC: Resources, Writing – original draft, Writing – review & editing. T-HH: Conceptualization, Data curation, Formal analysis, Funding acquisition, Investigation, Methodology, Project administration, Resources, Software, Supervision, Validation, Visualization, Writing – original draft, Writing – review & editing.
