# Supplementary material for: Transcranial burst electrical stimulation contributes to neuromodulatory effects in the rat motor cortex
Source: Front Neurosci. 2023 Dec 11;17:1303014. doi: 10.3389/fnins.2023.1303014 (PMC10749301; doi:10.3389/fnins.2023.1303014)
Supplement: Supplementary file 1 [file Data_Sheet_1.docx]

Supplementary Material


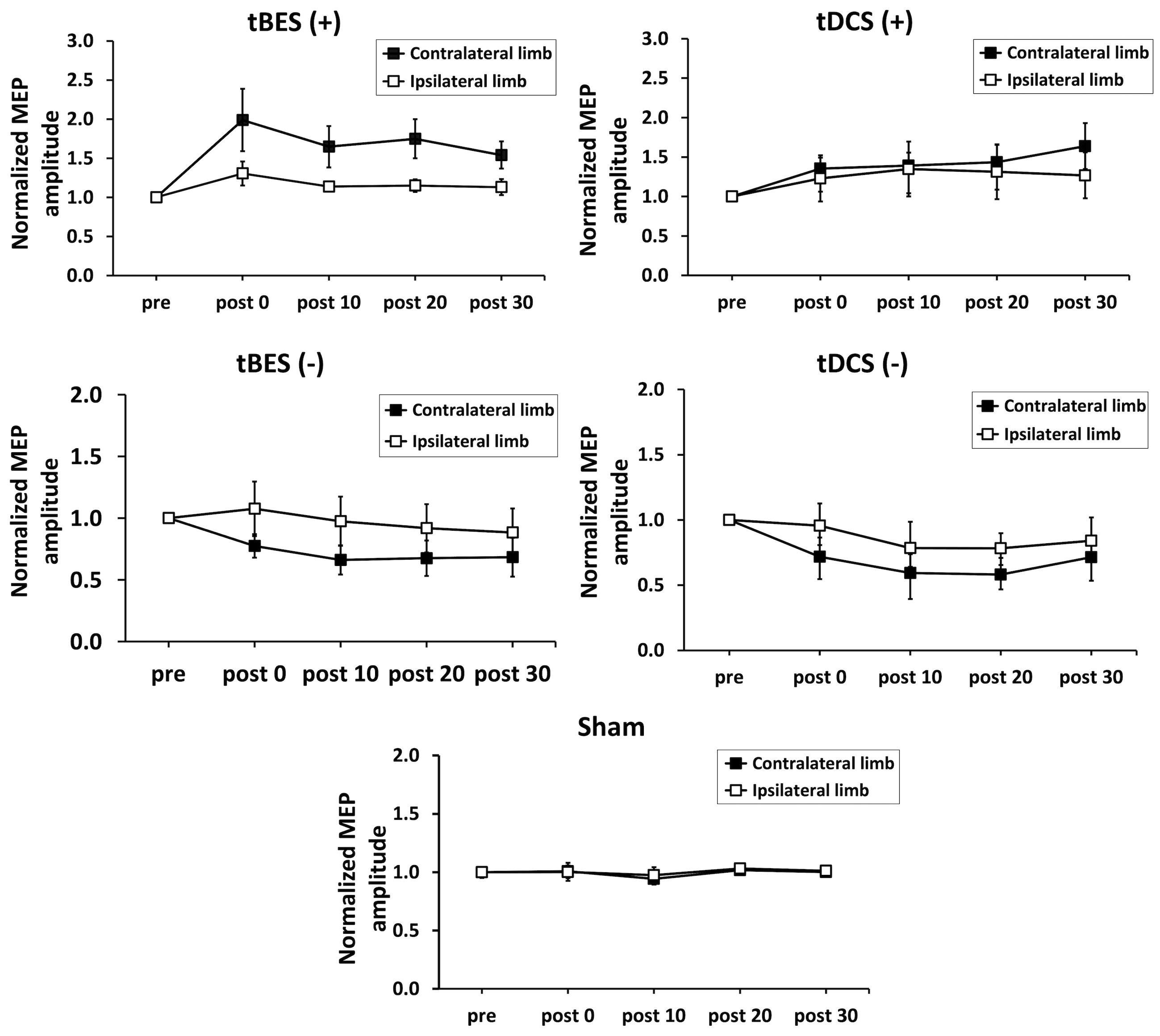


**Supplementary Figure 1.** The normalized motor-evoked potential (MEP) amplitudes for both the contralateral and ipsilateral limbs were assessed across the five intervention protocols (tBES+, tDCS+, tBES-, tDCS-, and sham). While changes in the amplitude of MEP induced by tBES (+), tDCS (+), tBES (-), tDCS (-) in the contralateral limb were greater than those in the ipsilateral limb, no statistical significance was observed. No changes in MEP were observed in either limb in the sham group.
